# Gender dynamics in digital health: overcoming blind spots and biases to seize opportunities and responsibilities for transformative health systems

**DOI:** 10.1093/pubmed/fdy180

**Published:** 2018-10-11

**Authors:** A S George, R Morgan, E Larson, A LeFevre

**Affiliations:** 1 School of Public Health, University of the Western Cape, Cape Town, South Africa; 2 Johns Hopkins Bloomberg School of Public Health, Johns Hopkins University, Baltimore, USA; 3 Faculty of Health Sciences, Cape Town, South Africa

**Keywords:** digital health, gender, health systems, gender dynamics

## Abstract

Much remains to ensure that digital health affirms rather than retrenches inequality, including for gender. Drawing from literature and from the SEARCH projects in this supplement, this commentary highlights key gender dynamics in digital health, including blind spots and biases, as well as transformative opportunities and responsibilities. Women face structural and social barriers that inhibit their participation in digital health, but are also frequently positioned as beneficiaries without opportunities to shape such projects to better fit their needs. Furthermore, overlooking gender relations and focussing on women in isolation can reinforce, rather than address, women’s exclusions in digital health, and worsen negative unanticipated consequences. While digital health provides opportunities to transform gender relations, gender is an intimate and deeply structural form of social inequality that rarely changes due to a single initiative or short-term project. Sustained support over time, across health system stakeholders and levels is required to ensure that transformative change with one set of actors is replicated and reinforced elsewhere in the health system. There is no one size prescriptive formula or checklist. Incremental learning and reflection is required to nurture ownership and respond to unanticipated reactions over time when transforming gender and its multiple intersections with inequality.

## Abstract (sp)

**RESUMEN:**

Resta mucho por hacer para que se consolide la salud digital y se reduzca la desigualdad, inclusive para el género. Basándose en la literatura y los proyectos SEARCH en este suplemento, este comentario destaca la dinámica de género clave en la salud digital, incluyendo los puntos ciegos y las parcialidades, también las oportunidades y responsabilidades transformativas. Las mujeres enfrentan barreras estructurales y sociales que inhiben su participación en la salud digital; pero también se las posiciona como beneficiarias sin oportunidades para formar tales proyectos de modo de ajustarse mejor a sus necesidades. Además, ignorar las relaciones de género y focalizarse en las mujeres aisladas puede reforzar, en lugar de abordar, las exclusiones de las mujeres en la salud digital y empeorar las consecuencias negativas no previstas. Si bien la salud digital brinda oportunidades para transformar las relaciones de género, el género sigue siendo una forma de desigualdad social muy personal y muy estructural que raramente cambia debido a una iniciativa simple o a un proyecto a corto plazo. La ayuda continua en el tiempo, presente en todas las partes interesadas y los niveles de los sistemas de salud se necesita para garantizar que el cambio transformativo con un grupo de participantes se replique y refuerce en todo el sistema de salud. No existe una fórmula o lista de control prescriptiva única. La reflexión y el aprendizaje gradual se necesitan para nutrir el control y para responder a las reacciones no previstas en el tiempo en relación a la transformación de género y sus intersecciones múltiples con la desigualdad.

**Palabras clave:**

salud digital, género, sistemas de salud, dinámica de género

## Abstract (fr)

**RÉSUMÉ:**

Il reste encore beaucoup à faire pour établir que la santé numérique maintient plutôt l’inégalité au lieu de l’éliminer, et cela touche le genre. En s’appuyant sur la documentation et les projets de recherche de ce supplément, ce commentaire met en évidence les principales dynamiques liées au genre dans le secteur de la santé numérique, y compris les zones d’ombre et les idées préconçues, ainsi que les occasions et responsabilités de transformation. Les femmes se heurtent à des obstacles structurels et sociaux qui les empêchent de participer à la santé numérique, mais elles sont également réduites, la plupart du temps, en bénéficiaires sans possibilité de concevoir de tels projets pour mieux répondre à leurs besoins. En outre, négliger les relations entre les sexes et mettre l’accent sur les femmes isolées ne peut que renforcer plutôt que résoudre les exclusions des femmes en matière de santé numérique tout en aggravant les conséquences négatives inattendues. Bien que la santé numérique offre la possibilité de transformer les relations entre les sexes, le genre constitue une forme d’inégalité sociale intime et profondément structurelle qui change rarement grâce à une initiative unique ou un projet à court terme. Un soutien accru dans le temps, par l’entremise des parties prenantes et des niveaux du système de santé, est nécessaire pour faire en sorte qu’un changement en profondeur impliquant un ensemble d’acteurs soit reproduit et renforcé ailleurs dans le système de santé. Il n’existe pas de formule ni de liste de vérification normalisées et universelles. Un apprentissage progressif et une réflexion sont nécessaires pour favoriser l’appropriation et réagir aux réactions imprévues au fil du temps lors de la transformation du genre et de ses multiples ramifications avec l’inégalité.

**Mots-clés:**

santé numérique, genre, systèmes de santé, dynamique liée au genre

## Introduction

Electronic, mobile and wireless technologies are near ubiquitous in low-, middle- and high-income countries. Their incorporation into health offers an unprecedented opportunity to strengthen health systems and improve health outcomes in innovative ways. Despite this potential, much remains to be done to strengthen how digital health affirms rather than retrenches inequality, including that involving gender.

Gender inequalities are often detailed as differences between girls and boys, women and men in binary and heteronormative ways. While gender analysis entails understanding these differences, it acknowledges the fluid and socially constructed nature of gender. It goes further to examine the power relations that shape the different identities, experiences and opportunities within and among different groups of women, men and gender-diverse people, or gender non-conforming people, including transgender and intersex people. This includes understanding how these varied forms of experiencing gender intersects with other social markers such as age, sexuality, disability, ethnicity, class and geographic location. It also entails identifying pathways for redressing such inequalities and transforming their underlying power relations.

Drawing from existing literature and experience working with a cohort of projects featured in this supplement and funded by Canada’s International Development Research Centre and under the collective title of ‘SEARCH’ (The ‘Strengthening Equity through Applied Research Capacity building in eHealth’ (SEARCH) cohort, funded by IDRC, supported research projects in Bangladesh, Burkina Faso, Ethiopia, Kenya, Lebanon, Peru and Vietnam. These projects examined if, how, and in what contexts digital health can meet key challenges faced in delivering quality health services and inform policy discourse to ensure that no one is left behind. In addition to the individual project results, analysis was conducted across the cohort on some cross-cutting issues, including gender dynamics.), this commentary outlines gender dynamics in digital health, including blind spots and biases. We go on to reflect on efforts to strengthen gender analysis within the SEARCH cohort. In doing so, we encourage seizing opportunities and responsibilities for realizing the transformational potential digital health hold for more equitable health systems.

## Understanding digital health gendered blind spots and biases

Given their disadvantage, girls and women are often targeted as key beneficiaries of programs. Yet they may continue to be missed if digital health is blind to gendered social relations that govern access to digital technologies. Women in low-income countries, while not a homogenous group, are overall far less likely to own or have independent control over mobile and wireless technologies than men.^1^ As a result, men can sometimes dominate digital health programs, even if they were primarily intended for women. In Uganda, men participated twice as much as women in an SMS-based HIV campaign and in the Democratic Republic of Congo over 80% of callers on a family planning hotline were men.^2,3^ It is crucial to meet men’s health awareness and needs, and to recognize marginalized gender dynamics and power relations among men given that they are also not a homogenous group. But this should not inadvertently widen pre-existing gender inequalities in access to information nor support men’s appropriation of programs designed for women.

In being blind to social relations, digital health interventions may not only miss key intended populations, but may also potentially further place them at risk. Many demand side digital health programs require that beneficiaries have access to mobile phones—a pre-requisite that can serve to exacerbate existing inequalities in the promotion of and access to health services. Digital health programs which provide mobile health information content to pregnant and postpartum women have the potential to increase women’s self-efficacy to breastfeed newborns or practice family planning. However, they may also threaten men and/or broader familial relationships if the messaging empowers practices in conflict with prevailing social norms.^4^ Supply-side digital health programs may offer the potential to improve the quality of care, through decision support or data capture. However, if individual patient records are not tracked or stored in a confidential manner, inadvertent disclosures can compromise women’s privacy and autonomy.^5,6^ Understanding existing gender dynamics and working with men, women and other key family and community members that replicate gender power relations within digital health programs can help to avoid such negative outcomes.

Not only do girls, women and other marginalized gender identities face structural and social barriers that inhibit their equal participation in digital health, they are also frequently positioned as beneficiaries of projects without opportunities to actively engage in and shape such projects to better fit their needs. Digital health programs must do more in foregrounding the voice and agency of marginalized populations in shaping program design and delivery strategies. In addition, rather than target girls and women as homogenous groups and in isolation from social context, given the repercussions in shifting gender power relations with community leaders, parents, in-laws, spouses and/or older siblings, it is imperative to consult and engage with these key gatekeepers for broader social and structural change to be realized.

## Seizing digital health opportunities to transform gender inequality

Despite gendered blind spots and biases, digital health has great potential to make a positive impact on gender relations. A systematic literature review^7^ found that digital health initiatives can improve couple communication, women’s decision-making, social status and access to health resources. The latter is particularly important for remote rural areas where there is a dearth of health professionals and services and for poor and marginalized communities for whom the cost of travel to reach health services is exorbitant. In Vietnam, minority ethnic women had limited knowledge and access to care. The text messages sent via the mMOM platform addressed some of these vulnerabilities.^8^ In Peru, given that husbands did not attend prenatal check-ups due to work obligations, women reported appreciating accessing and sharing information from trusted sources such as health providers through the Internet with their partners.^9^

Enhancing women’s autonomy enables them to be more involved in health seeking and health decision-making, both for themselves and other family members, and produces better health outcomes.^10^ Among the SEARCH cohort, receiving actionable health education via digital health bolstered the confidence of ethnic minority women in Vietnam. Being more informed and confident also improved their interactions and relationships with health providers.^8^ Future research should ideally explore intersectionality between gender, ethnicity, age, parity and marital status, among a range of contextually relevant social markers, for the women and family members involved in the project.

Apart from addressing gender dimensions on the demand side of health care services, digital health solutions also have implications for supply-side gender dynamics. By supporting supervision, data use, protocols for decision-making, training and better referral, digital health programs may foster innovative ways of enhancing the skills, competencies, social status and effectiveness of frontline workers, who are often women.^10^ In Peru, WawaRed, the integrated mobile phone system, enhanced the status of midwives and promoted greater equity among health workers in primary health centers as everyone could access the e-records.^9^ In Burkina Faso, mobile phone capabilities improved the community status of female health workers equipping them with new skills that enabled them a broader professional remit.^11,12^ Mobile phones also improved the social status of Health Extension Workers (HEWs) in Ethiopia and the improved data quality also motivated them further.^12^

## Gendered digital health responsibilities: minding gender gaps to transform health systems

The SEARCH cohort was designed with a number of cross-cutting areas of analysis within a developmental evaluation.^13^ Among these was a strong focus on gender and equity. As central parts of the SEARCH initiative, and given that IDRC’s emphasis on gender within their research funding and grant management, each SEARCH project had dedicated sections for gender analysis in their proposals. Moreover, analysis of research teams and initial engagement with them revealed existing capacity for gender analysis and responsiveness to strengthening consideration of gender within their work. Despite this clear intention and engagement, the mid-term review revealed this awareness and interest was not necessarily operationalized throughout research undertaken by the SEARCH cohort. Initial project responses were largely women centered without necessarily demonstrating engagement with the gendered power relations and social contexts of female beneficiaries, including their diverse family formations. For each of the seven projects, an external review, dialog and follow up was initiated. Additional resources and concrete ideas for action were provided to further prioritize and strengthen gender analysis in the projects and across the cohort as a whole.

As mentioned earlier, a critical first step is to understand existing gender inequalities and the power relations that underpin them. Consulting marginalized women and understanding their social context and relationships is vital to ensure that digital health addresses their needs and does not further harm them. However, addressing gender inequality does not mean working only with women. Men, leaders, decision makers and gatekeepers need to be involved and supportive of the overall digital health initiative, and also their gender transformative potential. In Lebanon, this entailed reaching out to men when they were available, which differed from when women were available.^14^ In Vietnam, programming was expanded beyond women to target messaging at men after identifying an interest among this demographic.^8^

It also entails exploring how gender intersects with other social stratifiers to influence experiences of marginalization, including among frontline workers. In Ethiopia, for example, researchers recognized how gender intersects with education, age and language ability to affect power dynamics between health workers and individuals across household, community and health system levels, including supervisors who were predominately older men.^12^

While digital health solutions may provide opportunities to transform gender relations, gender is an intimate and deeply structural form of social inequality that rarely changes due to a single initiative or short-term project. Sustained support over time, across health system stakeholders and levels is required to ensure that transformative change with one set of actors is replicated and reinforced elsewhere in the health system. In Burkina Faso, male community health workers were intentionally involved in the training of female participants, fostering a system of collaboration and support, rather than opposition. However, acceptance by husbands, who were not initially included in programming, took more time, and only occurred after their initial reluctance was identified and addressed by the research team.^11^

Positive gains without further review and support may fail to sustain transformation. In Ethiopia, while digital health solutions empowered one set of HEWs, inequalities between HEWs with mobile phones versus those without deepened. Due to the time required to input data, the digital health solution also displaced HEW time from other responsibilities and led to HEW out of pocket additional costs for downloading data.^12^

Given that digital health solutions are spread across diverse health system interfaces and levels, there is no one size prescriptive formula or checklist. Incremental learning and reflection is required to nurture ownership and respond to unanticipated reactions, particularly when shifting power relations underpinning gender inequality.^15^ Addressing gender in digital health projects requires sustained attention to avoid gender from fading away during implementation.^10^

## What next? Embracing gendered digital health toward positive transformation

The evidence base on how digital health addresses gender relations and supports gender equality is relatively thin. Changes in gender power relations and equality are rarely examined as an outcome, and even more rarely considered from the outset of projects. Furthermore, how gender intersects with other social stratifiers to influence experiences of marginalization is even more rarely explored. Studies often fail to sufficiently understand key stakeholders and their social networks, including program participants, supporting actors and gatekeepers. Data are often drawn from interviews with either women or men, at time without appreciating diversity within those categories, and rarely triangulate findings across women and men in the same project. Positive effects are often assumed or inferred by the authors, and negative effects are rarely measured.^7^

Ongoing monitoring, evaluation and research are required to understand more precisely the circumstances under which digital health solutions interact with gender intersecting with other social relations in partial or fully transformative ways, with intended or unintended consequences. Key questions include:

- Do girls, women and other marginalized gender groups have sufficient literacy, autonomy and ICT-access to effectively use digital health?
- In each context, what are other markers of social inequality and how do they interact with gender to affect digital health programs?
- How will use of digital health impact on and change existing gender power dynamics and relationships among key gender stakeholders, whether girls, boys, women, men, gender non-conforming people, other family or community members, at home, in communities, markets, or health services? What are the innovations in mapping and understanding these contextually configured and fluid power relations, particularly since perceived power may or may not translate into actual power?
- What kinds of engagement with those who enforce gender power relations, usually but not always men and boys, are necessary to transform gender relations in a positive way that increases both the effectiveness of digital health and improves the status of those marginalized by gender power relations, who are usually, but not always girls and women?

All digital health programs take place in a particular social, economic and political context. While informed by this context, they also provide the opportunity to transform some aspects of it. Recognizing this interactive dynamic between gender and digital health, we must support positive synergies between them, rather than allow gender inequalities to undermine digital health or have digital health further ignore and exacerbate gender inequalities. Gender inequalities operate in health systems at multiple levels and must be addressed at individual, community, program and policy levels if digital health is to achieve its full potential. Ongoing engagement with intended beneficiaries to understand and respond to the social relations and contexts they are embedded in, sustained over time with critical review and reflection, is vital for ensuring that digital health solutions actually support gender equality, rather than assume to do so.

## Supplementary Material

Supplementary Data

Supplementary Data
