# Supplementary material for: Gender dynamics in digital health: overcoming blind spots and biases to seize opportunities and responsibilities for transformative health systems
Source: J Public Health (Oxf). 2018 Oct 11;40(Suppl 2):ii6–ii11. doi: 10.1093/pubmed/fdy180 (PMC6294040; doi:10.1093/pubmed/fdy180)
Supplement: Supplementary Data [file fdy180_responses_to_reviewer_comments_20180914.docx]

| Reviewer 1 comments | Author responses |
| --- | --- |
| This was an extremely well written article that summarises the results of the SEARCH projects in the greater context of gender dynamics, with a recommendation of how to enhance this in future projects. I particularly appreciated the demand as well as supply side of the argument. There were however a couple of areas where I thought perhaps more analysis would be helpful. I have articulated these below. | Thank you for your thoughtful review and comments. |
| Many of these comments are not crucial for revision but I hope that perhaps they present some thought for discussion amongst the authors (and funders) that can enhance the assessment presented.  The authors mention often that gender is not just about men and women but rather embedded within a greater complexity of intersecting constructs such as age and social relations.  However, the commentary doesn’t articulate much about the intra-gendered complexities - Iin polygamous families the power of the older wife vs the youngest, and in traditional families the power of the mother in law for instance in making reproductive and other health choices for her son’s wife and children- and their relative agency to utilize ICT for the purposes intended. I believe that these are not absent in some of the contexts of the SEARCH projects. While such “gatekeepers” are mentioned, I found the commentary to be skewed towards the inter-gender power relations of men vs women and inadvertently limits the discussion on the wider ambit of gender dynamics and the impact on ICT agency and control. It would be great to hear more analysis (if any from the SEARCH projects or from the literature) on these other intersections mentioned but not elaborated on.  Furthermore, while I am sure this is not the intention, women and men come across as homogenous where in fact community research has demonstrated multiple times that within families and communities younger boys and girls perhaps are even more at risk of reduced agency than those older, even if within the same gender. This is alluded to but once again rests as a mere mention. | Thank you for these helpful insights and revised the text throughout. We have also added an example in paragraph 5 to illustrate potential complexities around the differential targeting of pregnant/ postpartum women within families and tried to bring more nu.  Initial research has shown that digital health in certain contexts is more accessible to younger generations than older ones, but further work needs to be done before drawing further conclusions.  While we recognize the importance of elaborating more on these and the other valuable themes noted, and have tried to accommodate their valuable real word nuances, word constraints prohibit their in-depth consideration here. |
| This comes back to the intersectionalities mentioned by the authors at several points with little exploration or comment. It would be interesting to know whether some of these intersectionalities are more pronounced in some contexts within the SEARCH projects more than others that may speak to the cultural influences on gender norms and dynamics. And if so, what are the implications of this in the “what next” section. | Exploration of how gender intersects with other social stratifiers within the SEARCH projects was limited and we are unable to say whether these intersectionalities are more pronounced in some contexts more than others.  We have however reiterated the importance of and need for this lens within the what next section (which is important for all contexts). |
| In the What next section I wonder if some of the key questions should also include a better understand of the stakeholder (both supportive as well as resistant) given the recognition of gatekeepers. I also wonder if one of the key activities or questions should be about mapping the social network to better unlock hidden power and gatekeepers within the communities in question. Perceptions of power are not often the same as real power and such activities or questions could be quite revealing. | These are helpful suggestions and ones that we concur with. We have added a sentence to the “What’s next” section emphasizing the need to improve understanding of key stakeholders and the need for methodologies that understand power relations. |
| Page 5 line 53...should read “interview” rather than “interviewing” Other key words beyond Gender would help with visibility of this article. Perhaps the various countries of the SEARCH projects, perhaps HPSR as well? | Thank you…the key words are unfortunately predetermined by the electronic submission format |
| Reviewer 2 comments |  |
| This paper is a much needed contribution in the world of information technologies. Thank you. My suggestions are request for revision are based on the fact that I know the first author is a leading gender/intersectionality expert and will be able to quickly make the needed revisions to strength the analysis of gender. I leave it to her expertise on how to incorporate the questions I have raised. | Thank you for your time and constructive feedback |
| On the one hand it is clear that the understanding and definition of gender is positioned on the “deeply structural form of social inequality” – yet there is a disconnect as there is a binary approach to gender and focus on women rather than power differentials across all gender identities. In the second paragraph of the introduction, the first line denotes gender in the cisgender sense rather than laying out the inequalities across the range of genders to include trans and inter-sex. While I understand the projects may have focused on women, there should be an explicit mention that it is the limitation of the gendered analysis. It is mentioned on page 5 lines 9-10 – should this not be made clear up front? This limitation is further compounded in the list of key questions identified on page 6. | This is a very valuable criticism of an inadvertent oversight. We have revised the manuscript to address the concerns while keeping within the overall word constraints. |
| The authors bring up the difficulty of addressing gendered inequalities in page 5, yet it would help if there were any learnings on why this was the case rather than just saying that more resources were introduced. | This goes to the point of how to support gender mainstreaming effectively as a political endeavor, which requires much more time and exploration than what this commentary can afford. |
| Another small point is the categorization of supply side on page 4 – is increasing social status a supply side category? Do you want to use a market descriptors of supply and demand in the context of a paper discussing power and health? | We take the reviewers point…this is mainstream health systems thinking. However also important to acknowledge there are also many different ways of seeing markets. Markets are imbued with power relations and are socially constructed. |
| These comments are made in the spirit of strengthening the gendered analysis of the paper. I trust that the authors will be able to make the revisions as they see fit given the data they have. | Thank you so much, we believe the revisions based on the constructive feedback have made the paper stronger. |
